# Higher total white blood cell and neutrophil counts are associated with an increased risk of fatal stroke occurrence: the Guangzhou biobank cohort study

**DOI:** 10.1186/s12883-021-02495-z

**Published:** 2021-12-02

**Authors:** Zhi-bing Hu, Ze-xiong Lu, Feng Zhu, Cao-qiang Jiang, Wei-sen Zhang, Jin Pan, Ya-li Jin, Lin Xu, G. Neil Thomas, Karkeung Cheng, Taihing Lam

**Affiliations:** 1 Guangzhou Twelfth People’s Hospital, Guangzhou, China; 2 School of Public Health, the University of Hong Kong, Pokfulam, Hong Kong; 3 School of Public Health, Sun Yat-sen University, Guangzhou, China; 4 Institute of Applied Health Research, University of Birmingham, Sun Yat-sen University, Birmingham, UK

**Keywords:** Stroke, WBC, Neutrophil, Ischaemic, Haemorrhagic, Cohort

## Abstract

**Background:**

Chronic inflammatory diseases are linked to an increased risk of stroke events. The white blood cell (WBC) count is a common marker of the inflammatory response. However, it is unclear whether the WBC count, its subpopulations and their dynamic changes are related to the risk of fatal stroke in relatively healthy elderly population.

**Methods:**

In total, 27,811 participants without a stroke history at baseline were included and followed up for a mean of 11.5 (standard deviation = 2.3) years. After review of available records, 503 stroke deaths (ischaemic 227, haemorrhagic 172 and unclassified 104) were recorded. Cox proportional hazards regression was used to assess the associations between the WBC count, its subpopulations and their dynamic changes (two-phase examination from baseline to the 1st follow-up) and the risk of fatal all stroke, fatal ischaemic stroke and fatal haemorrhagic stroke.

**Results:**

(i) Regarding the WBC count in relation to the risk of fatal stroke, restricted cubic splines showed an atypically U-curved association between the WBC count and the risk of fatal all stroke occurrence. Compared with those in the lowest WBC count quartile (< 5.3*10^9/L), the participants with the highest WBC count (> 7.2*10^9/L) had a 53 and 67% increased risk for fatal all stroke (adjusted hazard ratio [aHR] = 1.53, 95% confidence interval (CI) 1.16–2.02, *P* = 0.003) and fatal haemorrhagic stroke (aHR = 1.67, 95% CI 1.10–2.67, *P* = 0.03), respectively; compared with those in the lowest quartile (< 3.0*10^9/L), the participants with the highest NEUT count (> 4.5*10^9/L) had a 45 and 65% increased risk for fatal all stroke (aHR = 1.45, 95% CI 1.10–1.89, *P* = 0.008) and fatal ischaemic stroke (aHR = 1.65, 95%CI 1.10–2.47 *P* = 0.02), respectively. With the additional adjustment for C-reactive protein, the same results as those for all stroke and ischaemic stroke, but not haemorrhagic stroke, were obtained for the WBC count (4 ~ 10*10^9/L) and the NEUT count (the NEUT counts in the top 1% and bottom 1% at baseline were excluded). (ii) Regarding dynamic changes in the WBC count in relation to the risk of fatal stroke, compared with the stable group (− 25% ~ 25%, dynamic changes from two phases of examination (baseline, from September 1st, 2003 to February 28th, 2008; 1st follow-up, from March 31st 2008 to December 31st 2012)), the groups with a 25% increase in the WBC count and NEUT count respectively had a 60% (aHR = 1.60, 95% CI 1.07–2.40, *P* = 0.02) and 45% (aHR = 1.45, 95% CI1.02–2.05, *P* = 0.04) increased risk of fatal all stroke occurrence.

**Conclusions:**

The WBC count, especially the NEUT count, was associated with an increased risk of fatal all stroke occurrence. Longitudinal changes in the WBC count and NEUT count increase in excess of 25% were also associated with an increased risk of fatal all stroke occurrence in the elderly population.

**Supplementary Information:**

The online version contains supplementary material available at 10.1186/s12883-021-02495-z.

## Background

Stroke is classified mainly as ischaemic and haemorrhagic stroke [1]. With the high prevalence of comorbidities in developed Western countries, pre-existing chronic low-grade systemic inflammation has become a recognized characteristic of stroke pathophysiology [2]. Evidence now suggests that a chronic inflammatory response is associated with an increased risk of ischaemic [3, 4] and haemorrhagic [5] stroke. The total white blood cell (WBC) count, a plausible marker in the pathogenesis of chronic inflammation [6], is generally conducive to stroke incidence.

A higher WBC count on admission has been linked to poor outcomes, an increased risk of stroke mortality [7, 8], ischaemic stroke [9] and haemorrhagic stroke [10] in case-control studies. However, these WBC counts may be due to the stress reaction in acute patients with stroke [2], and it is not clear whether these higher WBC counts are linked directly to stroke death. On the other hand, a relatively high WBC count has been linked to stroke incidence, unfavourable functional outcomes and increased risks of fatal stroke [11–15] and ischaemic stroke [13, 15–20] in prospective cohort studies, although this is still controversial in the context of stroke [21], ischaemic stroke [22, 23] and haemorrhagic stroke [15, 19]. Similar associations have been shown between neutrophils, the largest WBC subpopulation, and stroke [8, 15], ischaemic stroke [15, 19, 20, 24–26] and haemorrhagic stroke [27]. However, different types of inflammation can result in increases in not only WBCs but also other indicators such as C-reactive protein (CRP). CRP, a controversial independent risk factor for stroke and an underlying acute inflammatory risk factor [2], has been reported to be a predictor of stroke [13, 28] and ischaemic stroke [23, 26, 29, 30]. Nevertheless, to date, no changes in the WBC count or its subpopulations have been reported to be linked to the risk of fatal stroke.

In previous work, we reported that a higher WBC count was associated with all-cause, CHD (coronary heart disease) and respiratory mortality [14], cardiovascular disease [31] and metabolic syndrome risk [32] in the Guangzhou Biobank Cohort Study (GBCS). Here, we aimed to systematically assess the relationships between the WBC count, its subpopulations and their changes and the risks of fatal all stroke, fatal ischaemic stroke and fatal haemorrhagic stroke among a relatively healthy elderly population in southern China.

## Methods

### Participants

All participants were recruited from a population of permanent residents aged 50 years or above in Guangzhou in southern China. Details of the GBCS, targetting an elderly population, have been reported previously [33]. The baseline (from September 1st, 2003, to February 28th, 2008) and follow-up information included a face-to-face computer-assisted interview by trained nurses on lifestyle [34], the family and personal medical history and assessments of anthropometrics, blood pressure and laboratory tests. Each participant had made an appointment in advance to ensure good health, was able to come the designated place by himself/herself and was able to sit and rest for at least half an hour before sampling and examination.

### Exposure indicators

The WBC count and subpopulation counts were performed with a blood cell counter (KX-21, Sysmex, Japan) in Guangzhou Twelfth People’s Hospital. The WBC, neutrophil (NEUT) and lymphocyte (LYM) counts were determined separately, while monocyte, eosinophil and basophil counts were determined automatically as a mixture (named MXDs). Fasting glucose, cholesterol, triglycerides, liver and kidney function and CRP were measured with an analyser (Cobas c-311, Roche, Switzerland). The hospital laboratory runs internal and external quality control procedures according to the China Association of Laboratory Quality Control.

### Study outcomes

Information on underlying causes of death up to December 31st, 2017, was obtained mostly via record linkage with the Guangzhou Centers for Disease Control and Prevention (GZCDC). Because there was no other information for stroke severity, infarct volume, site of lesion and infectious complications, fatal stroke occurrence was chosen as the primary outcome of this study. Death causes were coded according to the 10th revision of the International Classification of Diseases (ICD) as follows: I60 ~ I69 for stroke; I60.0 ~ I62.9 and I69.0 ~ I69.2 for haemorrhagic stroke; I63.0 ~ I63.9 and I69.3 for ischaemic stroke; and the other codes for unclassified stroke. When the death certificates were not issued by medical institutions, the causes were verified by GZCDC as part of their quality assurance programmed by cross-checking past medical history and conducting verbal autopsy by 5 senior clinicians from Guangzhou Twelfth People’s Hospital, the Universities of Hong Kong, China and Birmingham, UK.

### Potential confounders

To examine the extent to which baseline factors explained the associations of stroke, ischaemic stroke and haemorrhagic stroke, we included the factors in different models. Model 1 was a crude hazard ratio model without adjustment for any confounders. Model 2 contained multivariate adjustments including sex, age, education (primary and below, middle school, and college or above), occupation (manual, nonmanual, and others), smoking (never, former and current), alcohol consumption (never, former and current), International Physical Activity Questionnaire-assessed physical activity (inactive, moderate and active) [34], body mass index (BMI, defined as weight in kg÷eight in m^2^) [35], self-rated health (good, very good), hypertension, diabetes, dyslipidaemia, cancer, genitourinary disease (nephropathy, prostatic disease, and gynaecologic diseases), chest disease (chronic obstructive pulmonary disease, chronic bronchitis, emphysema, asthma, tuberculosis, and pneumonia) and the platelet count. Model 3 included CRP as a competing confounder in addition to the confounders in model 2.

### Statistical analysis

The WBC count was first analysed as a continuous parameter using a restricted cubic spline curve model with 3 knots at the 10th, 50th, and 90th percentiles of WBC counts. The WBC counts were also classified by quartiles. Categories of WBC, NEUT and LYM counts were defined as the following quartiles: 1st quartile (< 5.3*10^9/L), 2nd quartile (5.3–6.1*10^9/L), 3rd quartile (6.2–7.2*10^9/L) and 4th quartile (> 7.2*10^9/L) for the WBC count; 1st quartile (< 3.0*10^9/L), 2nd quartile (3.0–3.6*10^9/L), 3rd quartile (3.7–4.4*10^9/L) and 4th quartile (> 4.5*10^9/L) for the NEUT count; and 1st quartile (< 1.8*10^9/L), 2nd quartile (1.8–2.1*10^9/L), 3rd quartile (2.2–2.5*10^9/L) and 4th quartile (> 2.5*10^9/L) for the LYM count. For analysis on longitudinal WBC count changes, we chosed one follow up closest to baseline, thus only those who participated in the 1st follow-up (from March 2008 to December 2012) were included, and the follow-up period started from baseline (September 2003 to February 2008); an exposure period was therefore followed by the beginning of baseline. Two groups (±10 and ± 25%) were formed, with each group being drawn from those with two exposures and those who survived. Continuous variables are presented as the mean ± standard deviation, and categorical variables as presented as the frequency and percentage. The chi-squared and Fisher’s exact tests were used for categorical variables, and analysis of variance (ANOVA) and Kruskal-Wallis tests were used for continuous variables. Based on the results of the crude hazard ratio model analysis, a sensitivity analysis was conducted in which model 2 and model 3 were repeated for the participants with a normal range of WBC count (4 ~ 10*10^9/L) and with a NEUT count exclusion (The NEUT counts within the top 1% and bottom 1% at baseline were excluded. This exclusion was because of no normal range of NEUT count, and was to avoid cases with a significantly low or high NEUT count, though the number of such cases was small, and to avoid more loss of raw data). All analyses were performed using STATA (Version 14.0; StataCorp LP, College Station, TX, USA). All *p* values were 2 sided, and statistical significance was defined as *p* < 0.05; *p* values for trends in the models were calculated as ordinal scores from the 2nd, 3rd and 4th quartiles when taking the 1st quartile as the reference.

## Results

### Baseline characteristics

**Fig. 1:**
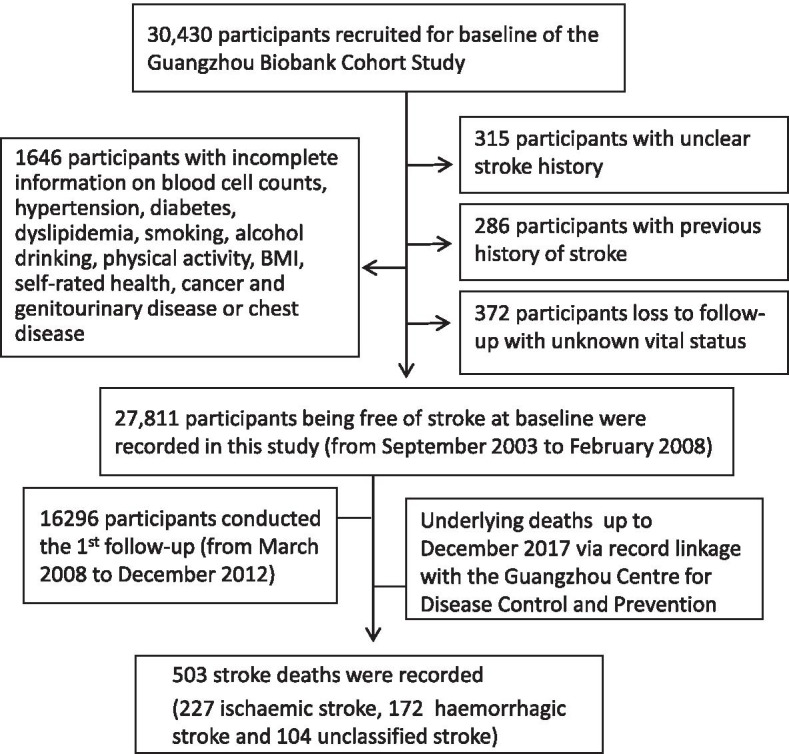
Flow diagram of participants selected for the analysis of this study

In total, 30,430 participants were screened. Among participant data exclusions, there were 286 because of a previous history of stroke, 315 because of an unclear stroke history, 372 because of loss to follow-up with unknown vital status, and 1646 because of incomplete information on the WBC, NEUT, LYM and platelet counts, hypertension, diabetes, dyslipidaemia, smoking, alcohol consumption, physical activity, BMI, self-rated health, cancer, genitourinary disease or chest disease. A total of 27,811 participants who were free of stroke at baseline were included in this study. After a mean follow-up time of 11.5 (standard deviation = 2.3) years with 320,859 person-years, 503 stroke deaths (227 ischaemic, 172 haemorrhagic and 104 unclassified) were recorded (Fig. 1).

The baseline characteristics of the participants are presented in Table 1. Compared to the population in the 1st WBC quartile, the population in the 2nd to the 4th quartiles had a higher proportion of men; were older; had a higher proportion of manual occupations; had a higher proportion of former or current smokers and drinkers; had higher proportions of individuals with BMIs ≥24 kg/m2, hypertension, diabetes and dyslipidaemia; had higher NEUT, LYM, and platelet counts and CRP levels; had a lower educational level; and had less physical activity, poorer self-rated health, and more cancer and genitourinary disease (all *P* < 0.001).

**Table 1:** Baseline characteristics by WBC quartiles of participants in the GBCS, 2003–2017 (*n* = 27,811)

Characteristics	Quartiles of WBC (*10^9/L)				*P* value trend
	1st (< 5.3)	2nd (5.3–6.1)	3rd (6.2–7.2)	4th (> 7.2)	
Number, n	6946	6912	7093	6860	
Sex, male(%)	1468 (21.1)	1767 (25.6)	2004 (28.3)	2392 (34.9)	< 0.001
Age (years)	61.1 ± 7.1	61.8 ± 7.2	62.3 ± 7.0	62.9 ± 7.0	< 0.001
Education (%)					< 0.001
Primary or below	2536 (36.5)	2861 (41.4)	3177 (44.8)	3406 (49.7)	
Middle school	3685 (53.1)	3405 (49.3)	3352 (47.3)	2932 (42.7)	
College or above	725 (10.4)	646 (9.3)	564 (8.0)	522 (7.6)	
Occupation					< 0.001
Manual	3277 (47.2)	3346 (48.4)	3551 (50.1)	3565 (52.0)	
Non-manual	2267 (32.6)	2262 (32.7)	2294 (32.3)	2130 (31.0)	
Others	1402 (22.2)	1304 (18.9)	1248 (17.6)	1165 (17.0)	
Smoking, n (%)					< 0.001
Never	6086 (87.6)	5822 (84.2)	5708 (80.5)	4909 (71.6)	
Former	530 (7.6)	596 (8.6)	682 (9.6)	714 (10.4)	
Current	330 (4.8)	494 (7.2)	703 (9.9)	1237 (18.0)	
Alcohol drinking, n (%)					< 0.001
Never	5016 (72.2)	4835 (70.0)	4965 (70.0)	4725 (68.9)	
Former	119 (1.7)	150 (2.2)	169 (2.4)	203 (3.0)	
Current	1811 (26.1)	1927 (27.8)	1959 (27.6)	1932 (28.1)	
Physical activity, *IPAQ*, n (%)					< 0.001
Inactive	649 (9.3)	505 (7.3)	560 (7.9)	541 (7.9)	
Moderate active	2844 (41.0)	2771 (40.1)	2855 (40.2)	2876 (41.9)	
Active	3453 (49.7)	3636 (52.6)	3678 (51.9)	3443 (50.2)	
Body mass index, kg/m^2^					< 0.001
< 18.5	562 (8.1)	295 (4.3)	199 (2.8)	190 (2.8)	
18.5–23.9	4119 (59.3)	3608 (52.2)	3339 (47.1)	2882 (42.0)	
24–27.9	1909 (27.5)	2429 (35.1)	2734 (38.5)	2785 (40.6)	
≥ 28	356 (5.1)	580 (8.4)	821 (11.6)	1003 (14.6)	
Self-rated health, n (%) (good/very good)	5724 (82.4)	5793 (83.8)	5899 (83.2)	5561 (81.1)	< 0.001
Hypertension, n (%)	1462 (21.0)	1748 (25.3)	2189 (30.9)	2417 (35.2)	< 0.001
Diabetes, n (%)	522 (7.5)	751 (10.9)	997 (14.1)	1359 (19.8)	< 0.001
Dyslipidemia, n (%)	5517 (79.4)	5694 (82.4)	5985 (84.4)	5828 (85.0)	< 0.001
Cancer, n (%)	180 (2.6)	137 (2.0)	122 (1.7)	100 (1.5)	< 0.001
GU disease, n (%)	2035 (29.3)	1873 (27.1)	1853 (26.1)	1644 (24.0)	< 0.001
Chest disease, n (%)	1060 (15.3)	1076 (15.6)	1039 (14.6)	1038 (15.1)	0.50
NEUT, *10^9/L	2.6 ± 0.76	3.3 ± 0.47	4.0 ± 0.95	5.4 ± 1.24	< 0.001
LYM, *10^9/L	1.7 ± 0.36	2.0 ± 0.41	2.2 ± 0.48	2.6 ± 0.67	< 0.001
Platelet, *10^9/L	203.6 ± 51.3	221.6 ± 57.7	233.9 ± 55.7	250.4 ± 65.8	< 0.001
CRP, mg/L	2.8 ± 2.4	3.1 ± 2.5	3.6 ± 2.8	4.2 ± 3.2	< 0.001
No. of all stroke deaths	89 (0.013)	98 (0.014)	136 (0.019)	180 (0.026)	< 0.001
No. of ischaemic stroke	42 (0.0060)	39 (0.0056)	66 (0.0093)	80 (0.012)	< 0.001
No. of haemorrhagic stroke	32 (0.0046)	37 (0.0054)	42 (0.0059)	63 (0.0092)	< 0.001

Hypertension: systolic blood pressure, ≥140 mmHg, diastolic blood pressure, ≤90 mmHg, medication or diagnosis; diabetes: fasting blood glucose ≥7, medication or diagnosis; dyslipidaemia: total cholesterol ≥5.2 mmol/L, triglyceride ≥1.7 mmol/L, low density lipoprotein ≥3.4 mmol/L, high density lipoprotein < 1.0 mmol/L, medication or diagnosis; *WBC* White blood cell, *CRP* C-reactive protein, *GU* Genitourinary disease (including nephropathy, prostatic disease, and gynaecologic diseases); chest disease (including chronic obstructive pulmonary disease, chronic bronchitis, emphysema, asthma, tuberculosis, and pneumonia)

### The WBC count in relation to the risk of fatal stroke occurrence

**Fig. 2:**
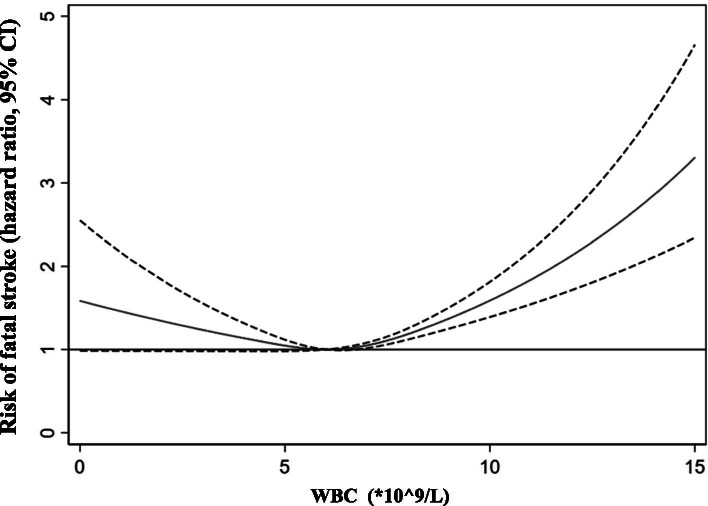
Association between the risk of fatal all stroke and WBC count on a continuous scale with restricted cubic spline curves based on Cox proportional hazards models in the GBCS followed for a mean 11.5 years. The solid blue line is the multivariable adjusted hazard ratio, with dashed lines showing 95% confidence intervals with three knots. A multivariate model adjusted for sex, age, education, occupation, diabetes, hypertension, dyslipidaemia, smoking, alcohol consumption, physical activity, body mass index, self-rated health, cancer, genitourinary diseases, chest disease and platelet count was used.

Our restricted cubic splines showed an atypically U-shaped association between the WBC count and the risk of fatal all stroke occurrence, and a WBC count of 6.3 *10^9/L was linked to the lowest risk of fatal all stroke occurrence after adjustments were made for potential confounders in model 2 (Fig. 2). Different risks of fatal all stroke occurrence were observed in the highest WBC quartile (aHR = 1.60, 95% CI 1.24–2.07, *P* < 0.001) and the lowest WBC quartile (aHR = 1.05, 95% CI 0.78–1.40, *P* = 0.76) when the 2^ed^ WBC quartile was taken as reference (Supplementary Table 1).

The left side of Table 2 shows the higher WBC counts in relation to the increased risk of fatal stroke. After adjustments for a series of factors, participants in the 4th WBC quartile (> 7.2*10^9/L) had increased risks of fatal all stroke (aHR = 1.53, 95% CI 1.16–2.02, *P* = 0.003) and fatal haemorrhagic stroke (aHR = 1.67, 95% CI 1.05–2.67, *P* = 0.03) but not fatal ischaemic stroke (aHR = 1.45, 95% CI 0.96–2.18, *P* = 0.08) compared to participants in the 1st WBC quartile (< 5.3*10^9/L). The participants in the 2nd, 3rd and 4th WBC quartiles had increasing risk trends for fatal all stroke (*P* < 0.001), fatal ischaemic stroke (*P* = 0.01) and fatal haemorrhagic stroke (*P* = 0.02). The middle of Table 2 shows the NEUT count in four quartiles. Significant associations with increased risks were fatal all stroke (aHR = 1.45, 95% CI 1.10–1.89, *P* = 0.008) and fatal ischaemic stroke (aHR = 1.65, 95% CI 1.10–2.47, *P* = 0.02). Unlike the WBC count, the NEUT count showed neither a higher risk (aHR = 1.14, 95% CI 0.74–1.75, *P* = 0.56) nor an increasing trend (*P* = 0.26) for fatal haemorrhagic stroke (Supplementary Fig. 1).

**Table 2:** Association between WBC counts and the risk of fatal stroke in the GBCS, 2003–2017 (n = 27,811)

	Quartiles of WBC (*10^9/L)				*P* value trend	Quartiles of NEUT (*10^9/L)				*P* value trend	Quartiles of LYM (*10^9/L)				*P* value trend
	1st (< 5.3)	2nd 5.3–6.1)	3rd (6.2–7.2)	4th(> 7.2)		1st(< 3.0)	2nd (3.0–3.6)	3rd (3.7–4.4)	4th (> 4.5)		1st(< 1.8)	2nd(1.8–2.1)	3rd(2.2–2.5)	4th (> 2.5)	
All stroke															
Person years	80,325	80,555	82,196	77,783		79,448	83,038	77,517	80,857		86,199	93,678	73,358	67,624	
per 10^5 person-years	110.8	121.7	165.5	231.4		109.5	116.8	152.2	248.6		178.7	156.9	140.4	146.4	
No. of deaths	89	98	136	180		87	97	118	201		154	147	103	99	
Model 1 (HR; 95% CI)	Ref.	1.09 (0.82–1.45)	1.48 (1.13–1.93)^b^	2.08 (1.61–2.68)^c^	< 0.001	Ref.	1.06 (0.79–1.41)	1.38 (1.05–1.82)^a^	2.25 (1.75–2.90)^c^	< 0.001	Ref.	0.88 (0.70–1.10)	0.78 (0.61–1.00)	0.82 (0.64–1.06)	0.07
*P* value		0.58	0.004	< 0.001			0.70	0.02	< 0.001			0.26	0.05	0.13	
Model 2 (HR; 95% CI)	Ref.	0.96 (0.72–1.28)	1.24 (0.94–1.64)	1.53 (1.16–2.02)^b**,**^	< 0.001	Ref.	0.87 (0.65–1.17)	1.06 (0.80–1.41)	1.45 (1.10–1.89)^b^	0.001	Ref.	1.01 (0.80–1.27)	0.92 (0.71–1.19)	0.95 (0.73–1.24)	0.56
*P* value		0.76	0.13	0.003			0.36	0.68	0.008		.	0.92	0.51	0.71	
Ischaemic stroke															
Person years	79,914	80,070	81,618	77,012		79,045	82,625	76,875	80,068		85,536	93,017	72,896	67,163	
per 10^5 person-years	52.6	48.7	80.9	103.9		46.8	54.5	61.1	122.4		86.5	77.4	52.1	64.0	
No. of deaths	42	39	66	80		37	45	47	98		74	72	38	43	
Model 1 (HR; 95% CI)	Ref.	0.92 (0.59–1.42)	1.52 (1.03–2.24)^a^	1.97 (1.36–2.86)^c^	< 0.001	Ref.	1.16 (0.75–1.78)	1.29 (0.84–1.99)	2.59 (1.78–3.79)^c^	< 0.001	Ref.	0.90 (0.65–1.24)	0.60 (0.41–0.89)^a^	0.75 (0.51–1.09)	0.03
*P* value		0.69	0.03	< 0.001			0.52	0.24	< 0.001			0.51	0.01	0.13	
Model 2 (HR; 95% CI)	Ref.	0.82 (0.53–1.28)	1.30 (0.87–1.94)	1.45 (0.96–2.18)	0.01	Ref.	0.95 (0.61–1.48)	1.00 (0.64–1.55)	1.65 (1.10–2.47)^a^	0.004	Ref.	1.05 (0.75–1.45)	0.71 (0.48–1.07)	0.89 (0.60–1.32)	0.24
*P* value		0.38	0.21	0.08			0.83	0.99	0.02			0.80	0.10	0.56	
Haemorrhagic stroke															
Person years	79,804	80,032	81,289	76,797		79,017	82,461	76,779	79,663		85,280	92,716	72,823	67,083	
per 10^5 person-years	40.1	46.2	51.7	82.0		50.6	37.6	48.2	82.8		64.5	43.1	57.7	52.2	
No. of deaths	32	37	42	63		40	31	37	66		55	40	42	35	
Model 1 (HR; 95% CI)	Ref.	1.14 (0.71–1.83)	1.27 (0.80–2.01)	2.02 (1.32–3.10)^b^	0.001	Ref.	0.74 (0.46–1.18)	0.94 (0.60–1.47)	1.61 (1.09–2.39)^a^	0.004	Ref.	0.67 (0.45–1.01)	0.89 (0.60–1.33)	0.81 (0.53–1.24)	0.55
*P* value		0.59	0.31	0.001			0.20	0.79	0.02			0.05	0.57	0.34	
Model 2 (HR; 95% CI)	Ref.	1.05 (0.65–1.70)	1.15 (0.71–1.85)	1.67 (1.05–2.67)^a^	0.02	Ref.	0.63 (0.39–1.02)	0.77 (0.49–1.22)	1.14 (0.74–1.75)	0.26	Ref.	0.77 (0.51–1.16)	1.03 (0.68–1.57)	0.94 (0.60–1.46)	0.94
*P* value		0.84	0.57	0.03			0.06	0.27	0.56			0.21	0.88	0.77	

Ref: reference; ^C^ *P* < 0.001, ^b^ *P* < 0.01, ^a^*P* < 0.05; model 1: a crude hazard ratio model without adjustment for confounders; model 2: a multivariate model adjusted for sex, age, education, occupation, diabetes, hypertension, dyslipidaemia, smoking, alcohol consumption, physical activity, body mass index, self-rated health, cancer, genitourinary disease (including nephropathy, prostatic disease, and gynaecologic diseases), chest disease (including chronic obstructive pulmonary disease, chronic bronchitis, emphysema, asthma, tuberculosis, and pneumonia) and platelet count

With the additional adjustment for CRP, the participants in the 4th WBC quartile had a significant association only for fatal all stroke (aHR =1.57, 95% CI 1.02–2.42, *P* = 0.04), but an increasing risk trend was evident for both fatal all stroke (*P* = 0.012) and fatal ischaemic stroke (*P* = 0.02) among 10,041 participants with normal WBC counts (4 ~ 10*10^9/L) (Left side of Table 3). The participants in the highest NEUT quartile had an increased risk for both fatal all stroke (aHR = 1.55, 95% CI 1.00–2.41, *P* = 0.05) and fatal ischaemic stroke (aHR = 2.47, 95% CI 1.24–4.93, *P* = 0.01), and an increasing risk trend was evident for both fatal all stroke (*P* = 0.009) and fatal ischaemic stroke (*P* = 0.004) among 9946 participants, with the NEUT count in the top 1% and bottom 1% being excluded; however, the higher NEUT count showed neither a significant association (*P* = 0.18) nor an increasing risk trend (*P* = 0.40) for fatal haemorrhagic stroke (Right side of Table 3).

**Table 3:** Association between WBC counts within normal range (4 ~ 10*10^9/L) and the risk of fatal stroke in the GBCS, 2003–2017 (*n* = 24,082)

	Quartiles of WBC (*10^9/L), n = 24,082				*P* value trend	Quartiles of NEUT (*10^9/L), *n* = 23,968				*P* value trend
	1st (< 5.3)	2nd 5.3–6.1)	3rd (6.2–7.2)	4th(> 7.2)		1st(< 3.0)	2nd (3.0–3.6)	3rd (3.7–4.4)	4th (> 4.5)	
All stroke										
Person years	64,170	74,055	75,331	64,985		61,162	76,172	71,150	67,682	
per 10^5 person-years	99.7	114.8	160.6	212.4		91.6	116.8	142.0	236.4	
No. of deaths	64	85	121	138		56	89	101	160	
Model 2 (HR; 95% CI)	Ref.	1.01 (0.72–1.40)	1.33 (0.97–1.82)	1.56 (1.13–2.14)^b^	0.001	Ref.	1.05 (0.75–1.48)	1.17 (0.84–1.64)	1.64 (1.19–2.26)^b^	< 0.001
*P* value		0.97	0.08	0.007			0.76	0.35	0.003	
Model 3 (HR; 95% CI)	Ref.	1.03 (0.66–1.60)	1.38 (0.90–2.10)	1.57 (1.02–2.42)^a^	0.012	Ref.	0.97 (0.61–1.54)	1.35 (0.87–2.09)	1.55 (1.00–2.41)^a^	0.009
*P* value		0.91	0.14	0.04			0.88	0.19	0.05	
Ischaemic stroke										
Person years	63,896	73,639	74,829	64,419		61,926	75,789	70,634	67,073	
per 10^5 person-years	53.2	48.9	78.8	97.8		45.2	55.4	60.9	116.3	
No. of deaths	34	36	59	63		28	42	43	78	
Model 2 (HR; 95% CI)	Ref.	0.81 (0.50–1.30)	1.21 (0.78–1.87)	1.29 (0.82–2.03)	0.08	Ref.	0.99 (0.61–1.60)	0.99 (0.61–1.60)	1.53 (0.97–2.42)	0.03
*P* value		0.38	0.39	0.27			0.96	0.96	0.07	
Model 3 (HR; 95% CI)	Ref.	0.92 (0.48–1.80)	1.48 (0.80–2.73)	1.77 (0.94–3.30)	0.02	Ref.	1.37 (0.67–2.82)	1.53 (0.75–3.13)	2.47 (1.24–4.93)^b^	0.004
*P* value		0.81	0.21	0.08			0.39	0.24	0.01	
Haemorrhagic stroke										
Person years	63,762	73,572	74,521	64,174		61,849	75,628	70,486	66,702	
per 10^5 person-years	29.8	39.4	51.0	67.0		35.6	35.7	41.1	75.0	
No. of deaths	19	29	38	43		22	27	29	50	
Model 2 (HR; 95% CI)	Ref.	1.23 (0.69–2.21)	1.56 (0.88–2.74)	1.91 (1.07–3.40)^a^	0.02	Ref.	0.87 (0.49–1.53)	0.94 (0.53–1.65)	1.51 (0.89–2.57)	0.06
*P* value		0.49	0.13	0.03			0.62	0.83	0.13	
Model 3 (HR; 95% CI)	Ref.	1.16 (0.56–2.41)	1.22 (0.59–2.53)	0.96 (0.43–2.14)	0.91	Ref.	0.61 (0.29–1.27)	0.93 (0.48–1.82)	0.60 (0.28–1.27)	0.40
*P* value		0.69	0.59	0.92			0.18	0.83	0.18	

Ref: reference; ^C^ *P* < 0.001, ^b^ *P* < 0.01, ^a^*P* < 0.05; model 2: a multivariate model adjusted for sex, age, education, occupation, diabetes, hypertension, dyslipidaemia, smoking, alcohol consumption, physical activity, body mass index, self-rated health, cancer, genitourinary disease (including nephropathy, prostatic disease, and gynaecologic diseases), chest disease (including chronic obstructive pulmonary disease, chronic bronchitis, emphysema, asthma, tuberculosis, and pneumonia) and platelet count; model 3: Model 2 + adjustment for CRP. WBC count analysis was conducted in 10,041 participants. NEUT count analysis was conducted in 9946 participants without stroke or a CVD history, and a NEUT count in the top 1% or bottom 1% was excluded.

Additionally, the LYM count showed only a decreased risk trend for fatal ischaemic stroke (*P* for crude HR =0.03). No significant association between fatal all stroke and the CRP level was observed (Table 4).

**Table 4:** Association between hs-CRP and the risk of fatal stroke in the GBCS, 2003–2017 (*n* = 11,601)

Quartiles of WBC (*10^9/L)	All stroke					Ischaemic stroke					Haemorrhagic stroke				
	1st (< 5.3)	2nd 5.3–6.1)	3rd (6.2–7.2)	4th (> 7.2)	*P-* value trend	1st (< 5.3)	2nd 5.3–6.1)	3rd (6.2–7.2)	4th (> 7.2)	*P*-value trend	1st (< 5.3)	2nd 5.3–6.1)	3rd (6.2–7.2)	4th (> 7.2)	*P* value trend
***Overall***															
Person years	34,882	36,186	35,976	35,665		34,608	35,832	35,644	35,269		34,485	35,765	35,499	35,123	
per 10^5 person-years	177.8	187.9	186.2	243.9		86.7	89.3	84.2	110.6		58.0	69.9	56.3	82.6	
No. of deaths	62	68	67	87		30	32	30	39		20	25	20	29	
Model 1 (HR; 95% CI)	Ref.	1.02 (0.72–1.44)	0.99 (0.70–1.40)	1.31 (0.94–1.81)	0.12	Ref.	0.98 (0.60–1.62)	0.90 (0.55–1.50)	1.19 (0.74–1.92)	0.53	Ref.	1.17 (0.65–2.11)	0.93 (0.50–1.73)	1.38 (0.78–2.44)	0.40
*P* value		0.92	0.96	0.11			0.95	0.70	0.48			0.60	0.82	0.27	
Model 2 (HR; 95% CI)	Ref.	0.93 (0.66–1.32)	0.85 (0.61–1.21)	1.04 (0.74–1.47)	0.87	Ref.	0.90 (0.54–1.48)	0.78 (0.47–1.31)	0.92 (0.56–1.53)	0.70	Ref.	1.07 (0.59–1.93)	0.81 (0.43–1.52)	1.16 (0.64–2.11)	0.80
*P* value		0.69	0.38	0.81			0.66	0.36	0.76			0.84	0.51	0.62	

Ref: reference; ^C^ *P* < 0.001, ^b^ *P* < 0.01, ^a^*P* < 0.05; model 1: a crude hazard ratio model without adjustment for confounders; model 2: a multivariate model adjusted for sex, age, education, occupation, diabetes, hypertension, dyslipidaemia, smoking, alcohol consumption, physical activity, body mass index, self-rated health, cancer, genitourinary disease (including nephropathy, prostatic disease, and gynaecologic diseases), chest disease (including chronic obstructive pulmonary disease, chronic bronchitis, emphysema, asthma, tuberculosis, and pneumonia) and platelet count

### WBC changes in relation to the risk of fatal stroke occurrence

The basic characteristics of the participants at the 1st follow-up are shown in Supplementary Table 2. Compared with that with a stable WBC count (from − 25 to 25%), the population with a WCB count gain (at > 25%) had higher proportions of manual occupations, former smokers and current drinkers; had higher proportions of moderate activity, BMIs ≥28 kg/m^2^, hypertension, cancer and chest diseases; lower proportions of other occupations, physical activity, and BMIs from 24 to 27.9 kg/m^2^; and lower WBC and NEUT counts (all *P* < 0.05).

Table 5 shows the association between the risk of fatal stroke and a change in the WBC count during the period from baseline (from September 2003 to February 2008) to the 1st follow-up (from March 2008 to December 2012). Compared to the stable participants, participants with WBC or NEUT count changes within 10% had no significant risk of fatal all stroke. Once the change reached 25% increased, a significant risk of fatal all stroke was present for both the WBC count (aHR = 1.60, 95% CI 1.07–2.40, *P* = 0.02) and NEUT count (aHR = 1.45, 95% CI 1.02–2.05, *P* = 0.04).

**Table 5:** Association between WBC count changes and the risk of fatal stroke in the GBCS, 2003–2012 (*n* = 16,296)

	All stroke			Ischaemic stroke			Haemorrhagic stroke		
	Loss (<−10%)	Stable (−10–10%)	Gain (> 10%)	Loss (<−10%)	Stable (−10–10%)	Gain (> 10%)	Loss (<−10%)	Stable (− 10–10%)	Gain (> 10%)
**WBC change**									
Person years	33,933	57,901	36,426	33,835	57,710	36,216	33,757	57,602	36,154
per 10^5 person-years	135.6	134.7	183.9	73.9	71.0	77.3	41.5	41.7	63.6
No. of deaths	46	78	67	25	41	28	14	24	23
Model 1 (HR; 95% CI)	1.01 (0.70–1.45)	Ref.	1.36 (0.98–1.89)	1.05 (0.64–1.73)	Ref.	1.08 (0.67–1.74)	0.99 (0.51–1.91)	Ref.	1.54 (0.87–2.73)
*P* value	0.96		0.07	0.85		0.76	0.97		0.14
Model 2 (HR; 95% CI)	0.93 (0.64–1.34)	Ref.	1.35 (0.97–1.88)	0.92 (0.56–1.52)	Ref.	1.06 (0.66–1.72)	0.94 (0.48–1.82)	Ref.	1.48 (0.83–2.63)
*P* value	0.70		0.08	0.75		0.80	0.85		0.18
**NEUT change**									
Person years	43,239	43,221	41,800	43,133	43,057	41,570	43,040	42,974	41,498
per 10^5 person-years	115.6	150.4	181.8	64.9	76.6	79.4	32.5	46.5	65.1
No. of deaths	50	65	76	28	33	33	14	20	27
Model 1 (HR; 95% CI)	0.76 (0.53–1.10)	Ref.	1.21 (0.87–1.69)	0.85 (0.51–1.40)	Ref.	1.04 (0.64–1.68)	0.69 (0.35–1.36)	Ref.	1.41 (0.79–2.52)
*P* value	0.15		0.25	0.52		0.89	0.28		0.24
Model 2 (HR; 95% CI)	0.72 (0.49–1.04)	Ref.	1.18 (0.85–1.65)	0.75 (0.45–1.24)	Ref.	1.00 (0.61–1.62)	0.69 (0.35–1.37)	Ref.	1.38 (0.77–2.46)
*P* value	0.08		0.33	0.26		0.99	0.29		0.28
	Loss (<−25%)	Stable (−25–25%)	Gain (> 25%)	Loss (<−25%)	Stable (−25–25%)	Gain (> 25%)	Loss (<−25%)	Stable (−25–25%)	Gain (> 25%)
**WBC change**									
Person years	7992	107,611	12,657	7972	107,225	12,565	7936	107,055	12,522
per 10^5 person-years	162.7	139.4	221.2	112.9	67.1	103.5	46.6	46.6	63.9
No. of deaths	13	150	28	9	72	13	3	50	8
Model 1 (HR; 95% CI)	1.18 (0.67–2.08)	Ref.	1.58 (1.06–1.37)^a^	1.72 (0.86–3.43)	Ref.	1.53 (0.85–2.76)	0.80 (0.25–2.57)	Ref.	1.38 (0.65–2.91)
*P* value	0.57		0.03	0.13		0.16	0.71		0.40
Model 2 (HR; 95% CI)	1.05 (0.59–1.85)	Ref.	1.60 (1.07–2.40)^a^	1.48 (0.74–2.98)	Ref.	1.58 (0.87–2.87)	0.77 (0.24–2.46)	Ref.	1.37 (0.65–2.92)
*P* value	0.86		0.02	0.27		0.13	0.65		0.41
**NEUT change**									
Person years	17,019	89,795	21,445	16,985	89,459	21,317	16,933	89,332	21,248
per 10^5 person-years	129.3	140.3	200.5	94.2	63.7	98.5	29.5	48.1	61.2
No. of deaths	22	126	43	16	57	21	5	43	13
Model 1 (HR; 95% CI)	0.92 (0.59–1.45)	Ref.	1.43 (1.02–2.03)^a^	1.49 (0.86–2.60)	Ref.	1.54 (0.94–2.55)	0.61 (0.24–1.53)	Ref.	1.29 (0.69–2.39)
*P* value	0.72		0.04	0.16		0.09	0.29		0.43
Model 2 (HR; 95% CI)	0.88 (0.56–1.38)	Ref.	1.45 (1.02–2.05)^a^	1.37 (0.79–2.40)	Ref.	1.59 (0.96–2.64)	0.61 (0.24–1.54)	Ref.	1.25 (0.67–2.34)
*P* value	0.57		0.04	0.27		0.07	0.30		0.49

Ref: reference; ^C^ *P* < 0.001, ^b^ *P* < 0.01, ^a^*P* < 0.05; model 1: a crude hazard ratio model without adjustment for confounders; model 2: a multivariate model adjusted for sex, age, education, occupation, diabetes, hypertension, dyslipidaemia, smoking, alcohol consumption, physical activity, body mass index, self-rated health, cancer, genitourinary disease (including nephropathy, prostatic disease, and gynaecologic diseases), chest disease (including chronic obstructive pulmonary disease, chronic bronchitis, emphysema, asthma, tuberculosis, and pneumonia) and platelet count

## Discussion

In this study, we found that both the WBC and NEUT were associated with the risk of fatal all stroke and that a higher NEUT count was associated with an increased risk of fatal ischaemic stroke. These associations were independent of age, sex, education, occupation, hypertension, diabetes, dyslipidaemia, smoking, alcohol consumption, physical activity, BMI, self-rated health, cancer, genitourinary disease, chest disease, platelet count and CRP.

An increasing number of studies on the relationship between the WBC count and stroke have focused mainly on the population at admission after stroke onset. Most of them support the notion that a higher WBC count is related to a poor outcome or mortality [8–10, 15, 26, 27, 36, 37], except for a few studies reporting disharmony with initial stroke severity [10, 15, 30, 38]. This indicates that inflammation arises together with stroke or that the stroke itself leads to leucocytosis or other poor outcomes. In a review [2], a series of biomarkers, including cytokines, the WBC count, CRP and interleukin 6 (IL-6), were shown to participate specifically in stroke progression [39]. When aimed specifically to address types of inflammation in mice, allergy (anaphylaxis) induced IL-10 and a corresponding response, while lipopolysaccharide stimulated various types of cells including WBCs to induce the release of a series of active molecules [40]. This is evidence for the effects of different types of inflammation on stroke progression.

We should discuss the corresponding relationship between the risk of fatal stroke occurrence and pre-existing chronic low-grade systemic inflammation. Because the GBCS collected a series of data from relatively healthy elderly individuals in South China, each appointment was made in advance to ensure the participant’s health and that each participant was able to come the designated place by himself/herself [32, 41]. To avoid missing important patterns in the relationship between the WBC count and incident fatal stroke, restricted cubic splines were employed, and the analysis showed a relationship between the WBC count on a continuous scale and a U-shaped risk of fatal all stroke occurrence, with high WBC counts being more related to an increased risk than low WBC counts. In the quartile analysis model, a higher WBC count linking the increased risk of fatal all stroke was verified again. In addition, after those with WBC counts at the highest and lowest ends of the range were excluded to avoid intervention during acute inflammatory reactions, our results became consistent with those of some previous reports [11, 13, 14]. The results were reaffirmed after further CRP adjustment, similar to reports from The Japan Collaborative Cohort Study [28] and The Glasgow Inflammation Outcome Study [42]. In contrast to the reports with incongruent factors [15–20], we found that the WBC quartiles showed an increasing risk trend for fatal ischaemic stroke; this weaker association may be due to our added adjustments for self-rated health, genitourinary disease, chest disease, the platelet count and CRP but lack of adjustments for total, HDL and LDL cholesterol, as well as fibrillation level. Nevertheless, a similar association for fatal haemorrhagic stroke disappeared after further adjustments.

As the largest subpopulation of WBCs, NEUTs play an important role in the major processes of atherosclerosis, thrombosis and stroke [43]. Our results are consistent with a few previous reports [15, 19, 20], though there are other conflicting reports [44–52], showing a higher NEUT count in relation to the increased risk for both fatal all stroke and fatal ischaemic stroke. When the WBC and NEUT counts for fatal stroke are taken into account, our findings suggest that the NEUT count is more conducive to predicting the risk of future fatal stroke occurrence. CRP has been reported to be an independent risk factor in clinical stroke [9, 26, 30]. Here, we observed no significant relationship between CRP and the risk of fatal all stroke (Table 4). This is likely because our analytic data was obtained from relatively healthy participants.

Individuals have different WBC background levels, which can fluctuate by 15% within 1 day [53]. Stroke events are related to chronic inflammation, while the WBC count can explain the immediate inflammation status well. Based on the baseline data and the first follow-up, we considered unhealthy conditions, random walks and native operation bias as being factors that were related to WBC variation. To guarantee the stability of WBC counts, each participant had an appointment made in advance, with enough time to rest for sampling and a fixed analyser measurement. We report first the risk of fatal all stroke in relation to changes in the WBC and NEUT counts in healthy elderly Chinese individuals. This indicates that an increasing WBC count or continuous chronic inflammation increases the risk of fatal stroke among older Chinese individuals. When WBC and NEUT counts and their dynamic changes are taken into account, it becomes clear that pre-existing chronic low-grade systemic inflammation plays an important role in future fatal stroke occurrence in the elderly population. This appears to be consistent with the existing body of literature highlighting the adverse cerebrovascular consequences of inflammation. Moreover, we observed an association between WBC count changes and the risk of fatal stroke occurrence in those with WBCs and NEUTs at low levels, although these levels were in the normal range regardless of baseline or the 1st follow-up. Therefore, clinicians should pay more attention to asymptomatic inflammation, especially the dynamic change in WBC counts, to curb the future risk of fatal stroke in a relatively healthy elderly population.

There are limitations in this study. First, we obtained only the death information via record linkage with the GZCDC. Our results, with death as the only outcome, are obviously weakened because of the lack of analysis on other clinical outcomes of stroke events. Second, among a series of potential confounders, inaccurate risk factors such as self-rated health may influence our results because of the high correlation with the objective indicators for health status [54]. Third, as the WBC count of each participant fluctuated, a longitudinal WBC change should be affected because of a native bias in every measurement, although we did more for each participant by making his or her appointment in advance, with sampling performed after an enough time was allowed for rest and conducting the measurement with a fixed analyser. Fourth, we enrolled only those who participated in the 1st follow-up in the study on longitudinal WBC changes, which introduces survivorship bias, and the bias was not considered by different types of analysis, such as group-based trajectory modelling or joint modelling of longitudinal and survival data. Fifth, the subjects could not represent Chinese individuals due to the limitations involving the general population in South China in this study. Finally, the small number of deaths limited the strength of this study to address fatal stroke, especially fatal ischaemic stroke and fatal haemorrhagic stroke.

## Conclusions

This first cohort study of relatively healthy Chinese individuals in one of the most economically developed cities in China found that higher WBC and NEUT counts were associated with an increased risk of fatal all stroke. Longitudinal WBC and NEUT count increases in excess of 25% were also associated with a significantly increased risk of fatal all stroke. Fatal stroke occurrence in China may forewarn the burden of pre-existing chronic low-grade systemic inflammation, especially in the elderly populations of large cities.

## Supplementary Information

**Additional file 1: Supplementary Table 1** Association between WBCs and fatal all stroke risk in the GBCS, 2003-2017 (n=27811). **Supplementary Table 2** Characteristics according to changes in the WBC count of participants in the GBCS (n=16296).

**Additional file 2: Supplementary Figure 1** Association between WBCs counts and the risk of fatal stroke among participants of the Guangzhou Biobank Cohort Study, 2003-2017 (n=27811).

## Data Availability

The datasets used during the current study are available from the corresponding author on reasonable request.

## Abbreviations

WBC: White blood cell
NEUT: Neutrophil
LYM: Lymphocyte
CRP: C-reactive protein
ICD: International Classification of Diseases
HR: Hazard ratio
aHR: Adjusted HR
cHR: Crude hazard ratio
CI: Confidence interval
GBCS: Guangzhou Biobank Cohort Study
GZCDC: Guangzhou Centers for Disease Control and Prevention
