# Supplementary material for: Higher total white blood cell and neutrophil counts are associated with an increased risk of fatal stroke occurrence: the Guangzhou biobank cohort study
Source: BMC Neurol. 2021 Dec 2;21:470. doi: 10.1186/s12883-021-02495-z (PMC8638334; doi:10.1186/s12883-021-02495-z)
Supplement: Supplementary file 1 — Additional file 1: Supplementary Table 1 Association between WBCs and fatal all stroke risk in the GBCS, 2003-2017 (n=27811). Supplementary Table 2 Characteristics according to changes in the WBC count of participants in the GBCS (n=16296). [file 12883_2021_2495_MOESM1_ESM.docx]

**Supplementary table 1** Association between WBCs and fatal all stroke risk in the GBCS, 2003-2017 (n=27811)

|  | Quartiles of WBC (*10^9/L) | | | |  | Quartiles of WBC within normal range (4~10*10^9/L) | | | |
| --- | --- | --- | --- | --- | --- | --- | --- | --- | --- |
|  | 1^st^ (<5.3) | 2^nd^ (5.3-6.1) | 3^rd^ (6.2-7.2) | 4^th^ (>7.2) |  | 1^st^ (<5.3) | 2^nd^ (5.3-6.1) | 3^rd^ (6.2-7.2) | 4^th^ (>7.2) |
| Person years | 80325 | 80555 | 82196 | 77783 |  | 64170 | 74055 | 75331 | 64985 |
| per 10^5 person-years | 110.8 | 121.7 | 165.5 | 231.4 |  | 99.7 | 114.8 | 160.6 | 212.4 |
| No. of deaths | 89 | 98 | 136 | 180 |  | 64 | 85 | 121 | 138 |
| Model 1 (HR, 95%CI) | 0.92  (0.69-1.23) | Ref. | 1.36  (1.05-1.76)^a^ | 1.92  (1.50-2.45)^c^ |  | 0.99  (0.72-1.38) | Ref. | 1.32  (1.00-1.75) | 1.55  (1.17-2.05)^b^ |
| *P* value | 0.58 |  | 0.02 | <0.001 |  | 0.97 |  | 0.05 | 0.002 |
| Model 2 (HR, 95%CI) | 1.05  (0.78-1.40) | Ref. | 1.30  (1.00-1.69)^a^ | 1.60  (1.24-2.07)^c^ |  | 0.98  (0.63-1.52) | Ref. | 1.34  (0.93-1.94) | 1.53  (1.05-2.22)^a^ |
| *P* value | 0.76 |  | 0.05 | <0.001 |  | 0.91 |  | 0.14 | 0.03 |

Ref: reference; ^C^ *P*<0.001, ^b^ *P*<0.01, ^a^*P*<0.05; model 1: a crude hazard ratio model without adjustment for confounders; model 2: a multivariate adjust model including sex, age, education, occupation, diabetes, hypertension, dyslipidemia, smoking, alcohol drinking, physical activity, body mass index, self-rated health, cancer, genitourinary disease(including nephropathy, prostatic disease, gynecologic diseases), chest disease(including chronic obstructive pulmonary disease, chronic bronchitis, emphysema, asthma, tuberculosis, pneumonia) and platelet count.

**Supplementary table 2** Characteristics according to changes in the WBC count of participants in the GBCS (n=16296)

| Characteristics | baseline | | | |  | the 1^st^ follow-up | | | |
| --- | --- | --- | --- | --- | --- | --- | --- | --- | --- |
|  | Loss (<-25%) | Stable (-25%-25%) | Gain (>25%) | *P v*alue |  | Loss (<-25%) | Stable (-25%-25%) | Gain (>25%) | *P* value |
| Number, n | 1052 | 13639 | 1605 |  |  | 1052 | 13639 | 1605 |  |
| Age (years) | 61.6±7.1 | 61.2±6.8 | 61.5±6.8 | 0.02 |  | 65.8±7.3 | 65.1±7.2 | 65.5±7.0 | 0.003 |
| Smoking, n (%) |  |  |  | <0.001 |  |  |  |  | <0.001 |
| Never | 828 (78.7) | 11267 (82.6) | 1313 (81.8) |  |  | 794 (75.5) | 10808 (79.2) | 1266 (78.9) |  |
| Former | 110 (10.5) | 1097 (8.1) | 172 (10.7) |  |  | 106 (10.1) | 1015 (7.5) | 148 (9.2) |  |
| Current | 114 (10.8) | 1275 (9.3) | 120 (7.5) |  |  | 152 (14.4) | 1815 (13.3) | 191 (11.9) |  |
| Alcohol drinking, n (%) |  |  |  | 0.09 |  |  |  |  | 0.86 |
| Never | 702 (66.7) | 9395 (68.9) | 1151 (71.7) |  |  | 567 (53.9) | 7379 (54.1) | 859 (53.5) |  |
| Former | 24 (2.3) | 295 (2.1) | 31 (1.9) |  |  | 374 (35.5) | 4765 (34.9) | 557 (34.7) |  |
| Current | 326 (31.0) | 3949 (29.0) | 423 (26.4) |  |  | 111 (10.6) | 1495 (11.0) | 189 (11.8) |  |
| Physical activity, *IPAQ*, n (%) |  |  |  | 0.07 |  |  |  |  | 0.21 |
| Inactive | 79 (7.5) | 1091 (8.0) | 146 (9.1) |  |  | 26 (2.5) | 274 (2.0) | 32 (2.0) |  |
| Moderate active | 408 (38.8) | 5330 (39.1) | 667 (41.6) |  |  | 223 (21.2) | 2731 (20.0) | 356 (22.2) |  |
| Active | 565 (53.7) | 7218 (52.9) | 792 (49.3) |  |  | 803 (76.3) | 10634 (78.0) | 1217 (75.8) |  |
| Body mass index, kg/㎡ |  |  |  | <0.001 |  |  |  |  | <0.001 |
| <18.5 | 54 (5.1) | 547 (4.0) | 85 (5.3) |  |  | 93 (8.8) | 766 (5.6) | 96 (6.0) |  |
| 18.5-23.9 | 556 (52.9) | 6819 (50.0) | 853 (53.1) |  |  | 563 (53.6) | 6560 (48.1) | 769 (47.9) |  |
| 24-27.9 | 359 (34.1) | 4927 (36.1) | 517 (32.3) |  |  | 319 (30.3) | 4858 (35.6) | 552 (34.4) |  |
| ≥28 | 83 (7.9) | 1346 (9.9) | 150 (9.3) |  |  | 77 (7.3) | 1455 (10.7) | 188 (11.7) |  |
| Self-rated health, n (% )  (good/very good) | 853 (81.1) | 11436 (83.8) | 1313 (81.8) | 0.01 |  | 924 (87.8) | 12242 (89.8) | 1426 (88.8) | 0.09 |
| Hypertension, n (%) | 259 (24.6) | 3576 (26.2) | 427 (26.6) | 0.48 |  | 486 (46.2) | 6732 (49.4) | 805 (50.2) | 0.11 |
| Diabetes, n (% ) | 131 (12.5) | 1638 (12.0) | 184 (11.5) | 0.73 |  | 164 (15.6) | 2189 (16.0) | 256 (16.0) | 0.92 |
| Dyslipidemia, n (%) | 829 (78.8) | 11412 (83.7) | 1270 (79.1) | <0.001 |  | 928 (88.2) | 12481 (91.5) | 1468 (91.5) | <0.001 |
| Cancer, n (% ) | 14 (1.3) | 249 (1.8) | 50 (3.1) | 0.001 |  | 35 (3.3) | 340 (2.5) | 66 (4.1) | <0.001 |
| GU disease, n (% ) | 309 (29.4) | 3815 (28.0) | 443 (27.6) | 0.57 |  | 416 (39.5) | 5236 (38.4) | 616 (38.4) | 0.76 |
| Chest disease, n (% ) | 173 (16.4) | 2034 (14.9) | 249 (15.5) | 0.36 |  | 247 (23.5) | 3103 (22.8) | 404 (25.2) | 0.09 |
| WBC, *10^9/L | 8.0±2.1 | 6.3±1.4 | 5.3±1.3 | <0.001 |  | 5.3±1.3 | 6.2±1.4 | 7.6±2.1 | <0.001 |
| NEUT, *10^9/L | 5.3±1.8 | 3.7±1.2 | 3.1±1.0 | <0.001 |  | 3.1±0.9 | 3.6±1.0 | 4.8±1.8 | <0.001 |

Hypertension: systolic blood pressure ≥140 mmHg, diastolic blood pressure ≤90 mmHg, medication or diagnosis; diabetes: fasting blood glucose ≥7, medication or diagnosis; dyslipidaemia: total cholesterol ≥5.2 mmol/L, triglyceride ≥1.7 mmol/L, low density lipoprotein ≥3.4 mmol/L, high density lipoprotein <1.0 mmol/L, medication or diagnosis; WBC: white blood cell; CRP: C-reactive protein; GU: genitourinary disease (including nephropathy, prostatic disease, and gynaecologic diseases); chest disease (including chronic obstructive pulmonary disease, chronic bronchitis, emphysema, asthma, tuberculosis, and pneumonia).
