# Supplementary material for: Higher total white blood cell and neutrophil counts are associated with an increased risk of fatal stroke occurrence: the Guangzhou biobank cohort study
Source: BMC Neurol. 2021 Dec 2;21:470. doi: 10.1186/s12883-021-02495-z (PMC8638334; doi:10.1186/s12883-021-02495-z)
Supplement: Supplementary file 2 — Additional file 2: Supplementary Figure 1 Association between WBCs counts and the risk of fatal stroke among participants of the Guangzhou Biobank Cohort Study, 2003-2017 (n=27811). [file 12883_2021_2495_MOESM2_ESM.pptx]

## Slide 1
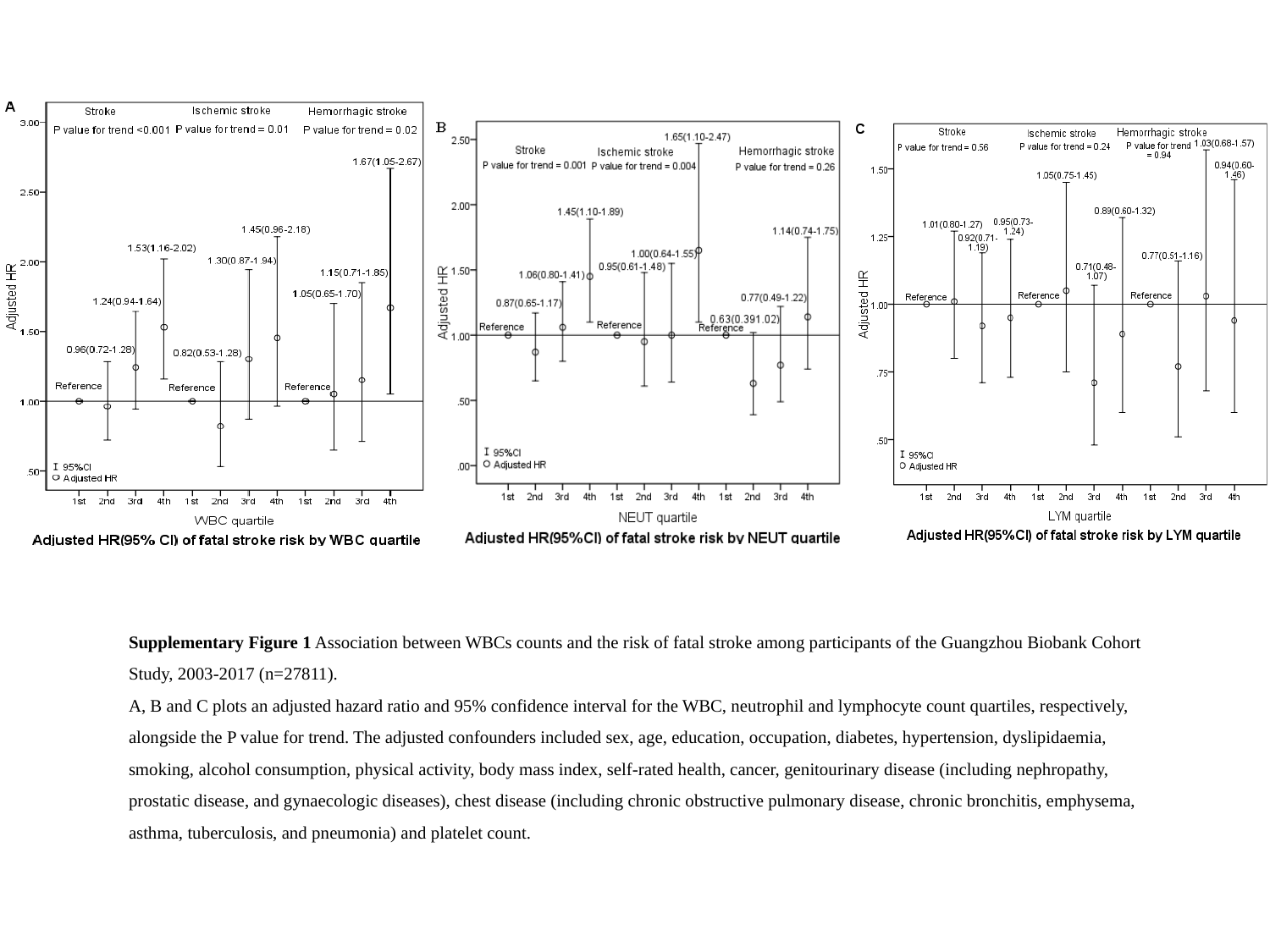

Supplementary Figure 1 Association between WBCs counts and the risk of fatal stroke among participants of the Guangzhou Biobank Cohort Study, 2003-2017 (n=27811).
A, B and C plots an adjusted hazard ratio and 95% confidence interval for the WBC, neutrophil and lymphocyte count quartiles, respectively, alongside the P value for trend. The adjusted confounders included sex, age, education, occupation, diabetes, hypertension, dyslipidaemia, smoking, alcohol consumption, physical activity, body mass index, self-rated health, cancer, genitourinary disease (including nephropathy, prostatic disease, and gynaecologic diseases), chest disease (including chronic obstructive pulmonary disease, chronic bronchitis, emphysema, asthma, tuberculosis, and pneumonia) and platelet count.
